# Study protocol for the St James's Hospital, Tallaght University Hospital, Trinity College Dublin Allied Researchers' (STTAR) Bioresource for COVID-19

**DOI:** 10.12688/hrbopenres.13498.1

**Published:** 2022-03-16

**Authors:** Laura O'Doherty, Stuart Hendricken Phelan, Nicole Wood, Sorcha O'Brien, Jacklyn Sui, Cian Mangan, Fergal Howley, Siobhan O'Regan, Noor Adeebah Mohamed Razif, Ciara Conlan, Ruth Argue, Samuel Holohan, Adam Dyer, Fara Salleh, Liam Townsend, Gerard Hughes, Colm Kerr, Derval Reidy, Alberto Sanz, Emma Connolly, Andrea Kelly, Emma Leacy, Conor Reddy, Siobhan Gargan, Eamon Breen, Heike Hawerkamp, Jean Dunne, Ignacio Martin-Loeches, Anne Marie McLaughlin, Aideen Long, Orla Shiels, Padraic Fallon, Martina Hennessy, Roman Romero-Ortuno, Ciaran Bannan, Anna Rose Prior, Ana Rakovac, William McCormack, Ross McManus, Seamus Donnelly, Colm Bergin, Mark Little, Clíona Ní Cheallaigh, Niall Conlon

**Affiliations:** 1 Department of Infectious Diseases, St. James's Hospital, Dublin, Dublin, Ireland; 2 Clinical Research Facility, St. James's Hospital, Dublin, Dublin, Ireland; 3 Department of Clinical Medicine, School of Medicine, Trinity Translational Medicine Institute, Trinity College Dublin, Dublin, Ireland; 4 Department of Immunology, St James's Hospital, Dublin, Ireland; 5 Department of Intensive Care Medicine, St James's Hospital, Dublin, Ireland; 6 Departments of Clinical Chemistry and Laboratory Medicine, Dublin 24 and School of Medicine, Tallaght University Hospital, Trinity College Dublin, Dublin, Ireland; 7 Department of Respiratory Medicine, Tallaght University Hospital, Dublin, Ireland

**Keywords:** COVID-19, coronavirus, SARS-CoV-2, biobank, clinical research, long covid, Dublin, Ireland

## Abstract

**Background**: The current coronavirus disease 2019 (COVID-19) pandemic began in Ireland with the first confirmed positive case in March 2020. In the early stages of the pandemic clinicians and researchers in two affiliated Dublin hospitals identified the need for a COVID-19 biobanking initiative to support and enhance research into the disease. Through large scale analysis of clinical, regional, and genetic characteristics of COVID-19 patients, biobanks have helped identify, and so protect, at risk patient groups The STTAR Bioresource has been created to collect and store data and linked biological samples from patients with SARS-CoV-2 infection and healthy and disease controls.

**Aim**: The primary objective of this study is to build a biobank, to understand the clinical characteristics and natural history of COVID-19 infection with the long-term goal of research into improved disease understanding, diagnostic tests and treatments.

**Methods**: This is a prospective dual-site cohort study across two tertiary acute university teaching hospitals. Patients are recruited from inpatient wards or outpatient clinics. Patients with confirmed COVID-19 infection as well as healthy and specific disease control groups are recruited.  Biological samples are collected and a case report form detailing demographic and medical background is entered into the bespoke secure online Dendrite database.

**Impact**: The results of this study will be used to inform national and international strategy on health service provision and disease management related to COVID-19. In common with other biobanks, study end points  evolve over time as new research questions emerge. They currently include patient survival, occurrence of severe complications of the disease or its therapy, occurrence of persistent symptoms following recovery from the acute illness and vaccine responses.

## Introduction

Coronavirus disease 2019 (COVID-19) is a novel, infectious, multi-system disease caused by severe acute respiratory syndrome coronavirus 2 (SARS-CoV-2) ^1^ The first case was confirmed in Ireland on the 26 ^th^ of February 2020 ^2^. On the 11 ^th^ of March 2020 the WHO declared COVID-19 to be a pandemic and since then this infectious disease and the effects of control measures have impacted on every aspect of normal life ^3^. Clinical features of the disease vary greatly, ranging from severe respiratory failure and death to mild cough or no symptoms ^4^. In the early stages of the pandemic clinicians from a range of disciplines in St.James’s Hospital (SJH) and Tallaght University Hospital (TUH), alongside researchers in Trinity College Dublin (TCD) identified the need for a biobanking initiative to support timely research into COVID-19. This led to the creation of the St James’s and Tallaght University Hospital and Trinity College Dublin Allied Researchers (STTAR) Bioresource for COVID-19.

**Image 1.**
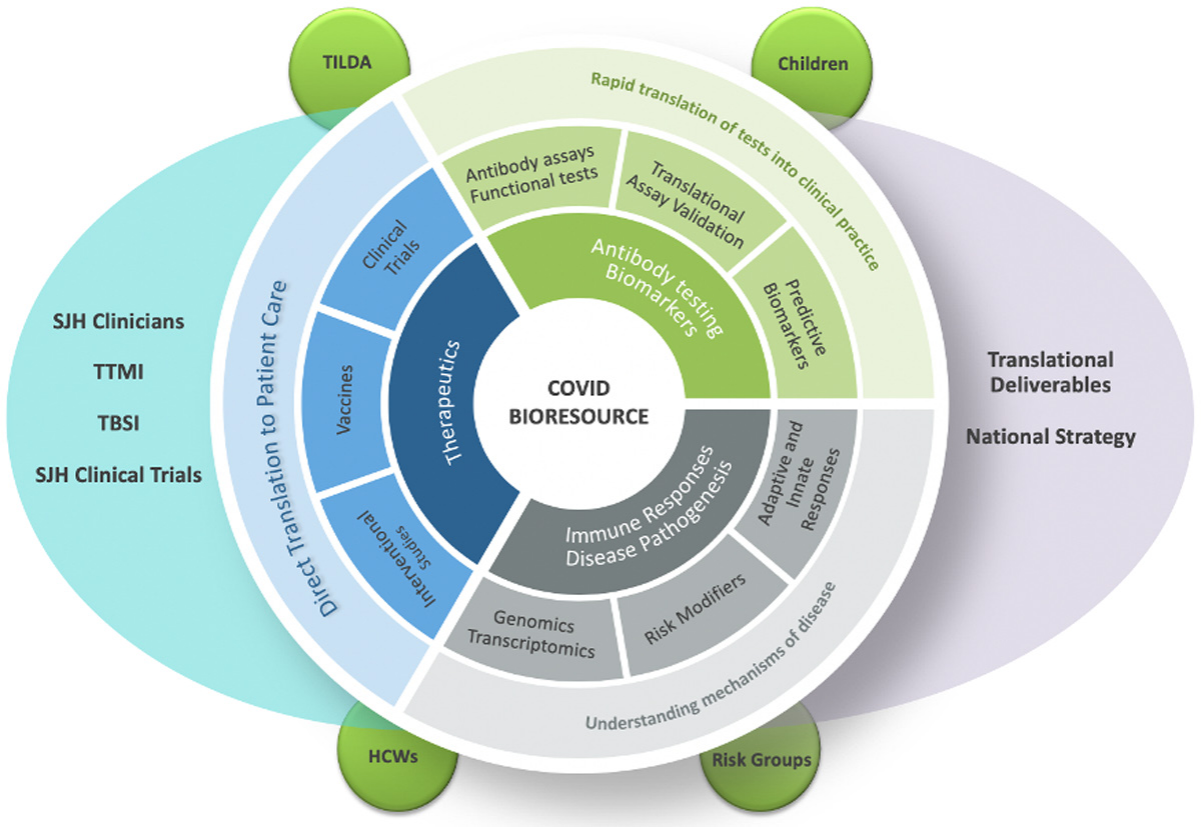
STTAR Bioresource Goals.

The aim of the STTAR Bioresource is to collect and store data and linked biological samples from St. James’s Hospital and Tallaght University Hospital patients with SARS-CoV-2 infection and healthy and disease controls and to use this resource within strict ethical and governance frameworks to support high quality research and inform national and international strategy on COVID-19 ( Image 1).

To achieve this goal, we have:

- Established and maintained a clinical database of COVID-19 infected patients
- Collected and stored biological samples (serum, plasma, PaxGene, leucocytes, DNA samples and saliva) and used these in concert with clinical data
- Compared differences in clinical characteristics and outcomes between those infected with COVID-19 and those in healthy and disease control groups.
- Built a powerful bespoke database linking granular clinical data with novel research data
- Shared coded samples and data with research groups to support local, national and international research initiatives.

As this novel disease continues to evolve, we have focused on understanding three aspects of COVID-19. The first is determinants of disease severity, including patient characteristics (e.g. age, sex, socio-economic status, co-morbidities), immunological features (e.g. immune cell phenotype) and alterations in proteins involved in coagulation. The second key area of interest for the group is patient recovery post COVID-19 disease and the study of prolonged symptoms. We aim to follow patients from admission through to follow up visits in the recovery stage of their illness. The third key area of interest is the immune response to COVID vaccines (including post-third dose/booster) across patient groups and healthy controls. Breakthrough infections post-vaccination or prior infection are a current focus of interest, and recruitment of these patients has been prioritised to enable analysis and research of these cases to begin without delay.

## Study protocol

### Study design

This is a prospective dual-site cohort study across two tertiary acute university teaching hospitals. The primary objective of this study is to build a biobank, to understand the clinical characteristics and natural history of COVID-19 infection with the long-term goal of research into improved disease understanding, diagnostic tests and treatments. We utilised a variety of existing infrastructures to support recruitment, clinical data collection, sample collection and storage, including the Department of Immunology, the Department of Infectious Diseases and the Wellcome Trust Clinical Research Facility at SJH and the Department of Respiratory Medicine in Tallaght University Hospital; and the Trinity Translational Medicine Institute (TTMI) to store and process these samples.

In common with other biobanks, study end points evolve over time as new research questions emerge. They currently include patient survival, occurrence of severe complications of the disease or its therapy, occurrence of persistent symptoms following recovery from the acute illness and vaccine responses.

### Research timeline

The start date for this study was April 2020 with the first recruit on the 27th April 2020. The duration of the bioresource has been open-ended and will evolve with time and need. Use of existing infrastructure and re-allocation of clinical and research staff allowed the early start of the study.

### Study setting

The STTAR Bioresource is situated in St James’s Hospital and Tallaght University Hospital in Dublin, Ireland. Our hospitals have a broad catchment area including parts of Kildare, South and West Dublin. Patients are referred for inpatient COVID-19 care by their general practitioner, emergency ambulance services or by self-presenting to the emergency department. Each hospital also receives inpatient transfers from secondary hospitals linked to them in the Dublin Midlands Hospitals Group ^5^.

In both sites, all patients are tested for COVID-19 on admission to hospital, regardless of their presenting illness. Staff members are also tested by the hospital if they develop symptoms suggestive of COVID-19 infection or in the case of hospital outbreaks. Moreover, patients may be referred to the hospital ‘long COVID’ follow-up outpatient clinic, for example from general practitioners (GPs) or following discharge after an admission for COVID-19 infection. These three patient groups – inpatients, hospital staff, and ‘long COVID’ outpatients as well as healthy and disease controls– were eligible for recruitment and inclusion in the Bioresource study population.

## Study population

### People with acute COVID-19

Inpatients with polymerase chain reaction (PCR) confirmed SARS-CoV-2 in the two recruiting acute hospital sites are invited to take part. Hospital staff with confirmed diagnoses of COVID-19 disease are also recruited. Further samples and data are collected at clinical follow-up in post-COVID clinics.

### Control group

Staff, patients, and members of the public who do not have acute COVID-19 are invited to participate. In order to facilitate effective matching to cases, controls from a range of ages and varying socio-economic status are sought. Control patients’ COVID-19 infection and vaccination status are recorded. Disease controls will be recruited from patients admitted with other respiratory infections (e.g. influenza).

### Other specific disease groups

Patients with polymerase chain reaction (PCR) confirmed SARS-CoV-2 infection with underlying complex immunodeficiency disorders and vasculitis are invited to take part in smaller subgroup studies. Their results are compared to the general COVID-19 positive study group and to the control group. There are no specific exclusion criteria.

## Sample and data collection

### Time points

**Image 2.**
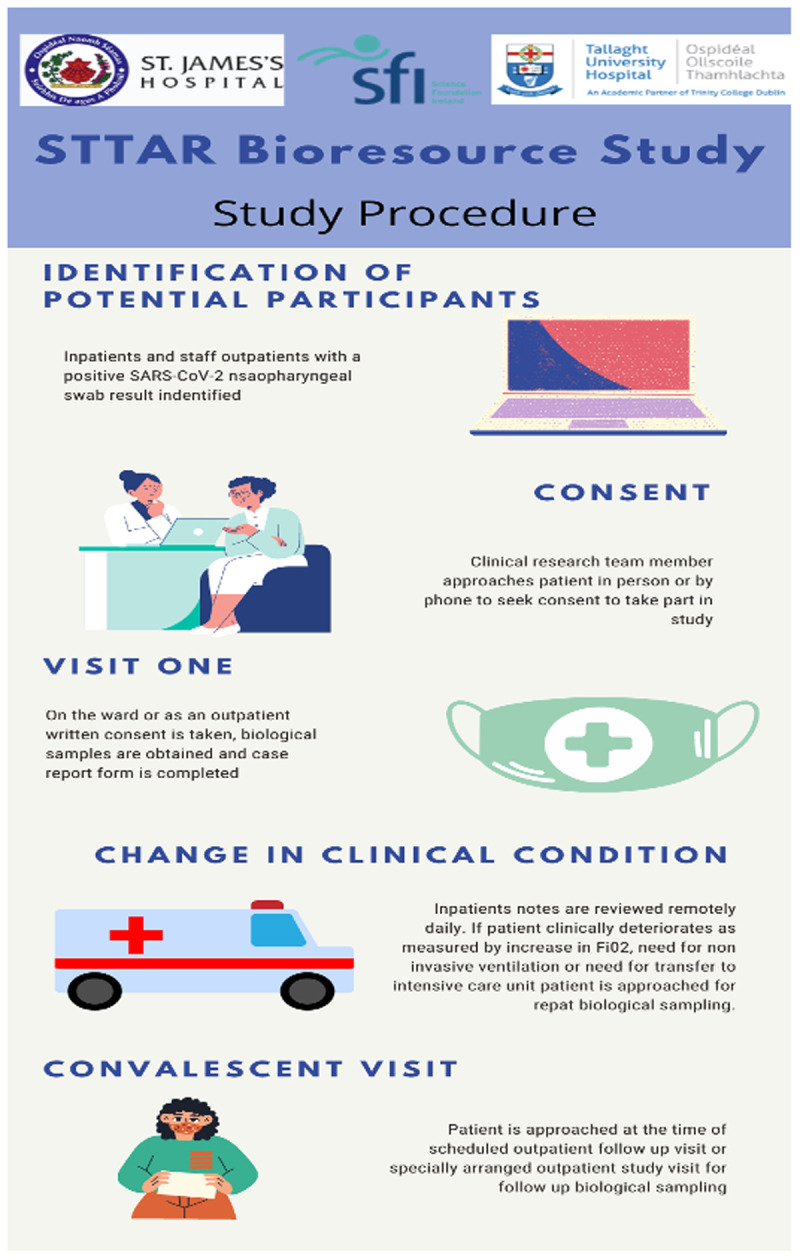
STTAR Bioresource Procedures Infographic.

A practical approach to recruitment was put in place during the various waves of the pandemic. It is acknowledged by the study team that there is value in both samples and high-quality patient data. In general, samples are taken the morning following diagnosis or admission during the routine phlebotomy round. The study team monitors the patient record and where there is a clinical deterioration marked by an increase in Fi02 or a requirement for additional ventilatory support or intensive care assessment, a second sample set is sought. Further samples at discharge or convalescence are sought. For practical reasons samples at each time point are not always available or required. Emphasis on the relative importance of each time point is dictated by the steering group in response to the pandemic environment. Convalescent patients are also recruited from clinics during attendance for routine clinical care ( Image 2).

### Sampling protocol: patients with incident COVID-19

New patients with positive SARS-CoV-2 tests are identified by the STTAR Bioresource Study team from daily internal communications of new positive SARS-CoV-2 PCR tests and COVID-19 ward admissions. Patients are approached for informed consent and provided with the STTAR Bioresource patient information leaflet. The project and use of the samples are explained in detail, with a specific focus on genetic aspects of the study. All patients are given an opportunity to review documentation and ask questions prior to signing the informed consent form. Patients are made aware that there are no consequences to not being involved in the study, informed that they can specifically opt out of genetic aspects of the study and that they can withdraw their data and samples from the study at any time. In cases where informed consent from a patient is not possible, next of kin assent is sought in line with our ethics approvals. If/when they regain capacity, we will approach them for retrospective consent.

A unique bioresource study ID is assigned to each consented patient and recorded in recruitment logs. Completed paper informed consent forms for both hospital sites are held centrally and securely in the Wellcome Trust Clinical Research Facility.

To minimise patient discomfort and reduce the numbers of contacts, study blood samples are taken at the same time as routine clinical samples. A case report form (Appendix A, Case Report Form) detailing demographic and medical background is completed and saliva samples taken by the research nurse obtaining consent (Appendix B, Informed Consent Form). This data is then entered to the secure Dendrite database. Blood samples in SJH are transported to the central processing lab after phlebotomy, where they are retrieved directly by a member of the bioresource team, for immediate processing in the TTMI laboratory. In TUH, blood and saliva samples are taken by the TUH bioresource research nurse and placed in a morning courier to TTMI. If genetics is not consented to, Paxgene samples are not taken from the patient.

### Withdrawal procedure

If the patient decides to withdraw from the study at any stage, the research team member documents this decision clearly in the patient’s medical notes and CRF detailing the reason if known. A withdrawal form is then completed.

Participants have the following options:

○ No further access: This means that no further data or samples will be collected. The participant will no longer be contacted. However, the Registry and Biobank will still have permission to use, store and share information and samples collected up until this date.
○ No further use: This means that no further data or samples will be collected. The participant will no longer be contacted. Samples held by the Biobank will be destroyed. Data held on the patient will be deleted. Researchers who have received samples and data will be contacted to request that unused samples and data be destroyed. Research results from data that has been analysed will continue to be used.

### Sample processing

**Image 3.**
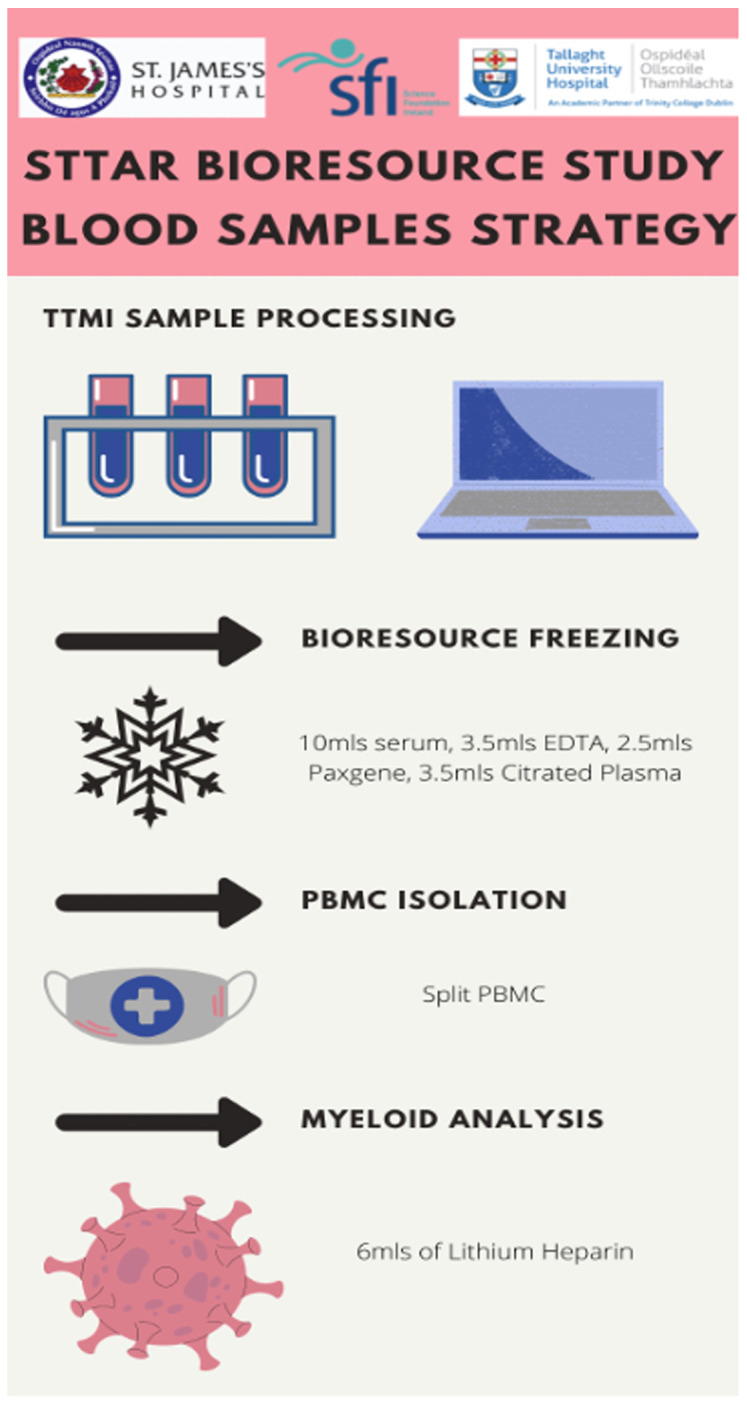
STTAR Bioresource Study laboratory sample strategy.

Samples are received by the STTAR team in a bag labelled ‘COVID BIORESOURCE’. Each bag contains 6 sample types and 8 tubes – the maximum volume collected is 46mls (see Table 1 and Image 3).

**Table 1.** Summary of samples and their storage.

Sample Type	Test Request	Sample processing	Sample Storage
10 mls Serum	IL-6	Centrifuged	4–6 Aliquots serum stored @ -80°C
3.5 mls Citrated	Coag	Centrifuged	3–5 Aliquots plasma stored @ -80°C
3.5 mls EDTA	TBNK	Centrifuged	3–5 Aliquots plasma stored @ -80°C, store original tube with remaining packed cells at -20°C for short term and transferred to-80°C for long term.
PAXGENE	None	None	Labelled Rack in -80°C (Left at RT for minimum of 2 hours, then for minimum O/N, followed by transfer to -80°C)
6mls Lithium Heparin	None	None	Sample to be collected by TTMI staff for processing by DECOMPRESS protocol or fresh use
18mls Lithium Heparin (remaining)	None	SEPMATE	Sample to be collected by TTMI staff for PBMC separation

### Safety

- SARS-CoV-2 has been classified as a risk group 3 biological agent. Laboratories carrying out non propagative research and development work can, subject to risk assessment, carry out work at minimum containment level 2 ^6^.
- Following local risk assessment the STTAR processing team work at containment level 3 in a CL2 facility. Periodic review of the risk assessment is carried out as delegated by the appropriate Health and Safety Committee.

### Process

- For samples 1–3, 4 – 6 aliquot tubes (0.5 ml Sarstedt micro tubes) are labelled with Study ID, timepoint, sample type and aliquot number.
- EDTA spun pellet and RNA Paxgene tubes are simply labelled with the Study ID and Timepoint number as they remain in their original tubes.
- Samples 1–3 are centrifuged @ 3,400 RPM @ room temperature for 10 minutes and the plasma / serum stored in Sarstedt 0.5 ml Micro tubes with cap 72.730.007. 250–300 microliters of sample are added to each labelled micro- tube
- All Aliquots and PAXGENE and EDTA are labelled with Study ID and entered into the biobank

- Functional Studies
  ○ Samples for functional studies are collected from TTMI by study staff
  ○ 6mls Lithium Heparin are processed as per the relevant protocol from the investigator lab for fresh sample analysis
  ○ The remaining (18mls Lithium Heparin) are processed using SepMate – PBMC is separated and split into one aliquot for immediate functional studies (if needed) and one aliquot for freezing.

### PBMC isolation using SepMate method

- 15mL of Lymphoprep is loaded into the bottom of SepMate 50 tube.
- Blood is pooled from collection tubes into 50mL tube and diluted 1:2 with PBS.
- The diluted blood (up to 35mL) is overlayed on lymphoprep while keeping the tube upright and then spun at 1200g for 10mins with break on.
- The top layer is then poured into a new 50mL tube and suspension is spun at 400g for 7mins with break on.
- The supernatant is discarded, and pellet resuspended in 10mL PBS (small sample taken for counting) and spun again at 400g for 7mins with break on.
- Supernatant is again discarded and pellet resuspended in appropriate volume of cryoprotective media (10%DMSO/90%heat inactivated FBS).
- Samples are divided into aliquots (1ml × 1×10 ^7^ cells/ml) and labelled with study ID for immediate use or for cryopreservation

### Cryopreservation

All steps should be performed immediately with minimal pipetting.

1. Prior to centrifuging cells for cryopreservation the number of vials to be stored down and the amount of freezing media required to achieve 1mL volumes at a final cell concentration of 1 x 10 ^7^ cells/mL is calculated.
2. All vials are labelled with liquid nitrogen safe labels with the study ID, sample type and vial number.
3. Freezing media is stored at 2–8°C until time of use.
4. Samples are centrifuged at 400g for 7 minutes, the supernatant discarded and tube gently flicked to resuspend the cell pellet.
5. 1mL of cold cryoprotective media (10%DMSO/90% heat inactivated FBS) (2–8°C) per 1 × 10 ^7^ cells is added and gently mixed.
6. 1mL aliquots are placed into labelled cryovials and then into the freezing container (at an appropriate temperature) which is then stored at -80°C at the TTMI in the STTAR Bioresource Biobank.

### Documentation in sample spreadsheet

Study number, sample type, aliquot number, position and row are documented in the COVID BIORESOURCE 2020 spreadsheet. Information on all samples received for processing and the aliquots generated, including Study ID, aliquot ID, processing date and location of aliquot storage are logged on the STTAR Biobank Patient Recruitment and Sample Log.

## Data and sample access

Applications for access to clinical data or samples from the STTAR Bioresource are considered by a designated committee and granted for specific reasons. Successful applications have been made to date by researchers investigating COVID-related anosmia, ‘long COVID’ symptoms and COVID-19 patients’ cardiovascular risk profiles. Applications are discussed by the STTAR steering group and either approved, held pending further information or rejected. There is a process for appeal in the context of sample rejection. The STTAR steering group makes its decisions based on the quality and feasibility of an application. Applications for biological samples are also assessed with reference to efficient use of the finite sample resource and avoidance of duplication of research effort.

### Dispensing of samples from biobank

TTMI biobank staff are notified by the Steering committee Chair that a sample request has been approved and the samples are dispensed to the recipient. All samples dispensed from the biobank are logged on STTAR COVID-19 Biobank Patient Recruitment and Sample Log and the following information is recorded:

- Recipient of sample
- Date of dispensing
- Aliquot ID
- Volume of sample given

These are interim processes pending implementation of Module Bio Biobank Information Management System *.*

### Clinical data collection

Patient characteristics (e.g. age, sex, level of education, co-morbidities, pre-admission medication) and features of COVID-19 infection (e.g. date of symptom onset, oxygen requirement, COVID-19 treatment, requirement for admission to intensive care, radiological findings, results of blood tests) are extracted from the electronic patient record in SJH and from the paper medical chart in TUH by clinical research nurses and/or clinical research fellow after completion of the acute inpatient episode. A subset of variables are recorded for the date of blood sampling for storage in the bioresource including symptoms, diseases severity oxygen requirement and medication. and (e.g. symptomatology, disease severity) Peak severity is defined using the WHO clinical progression score. Clinical data quality control is done by the PIs on 10% of cases selected at random.

The case report form (Appendix A) is completed by the research nurse on paper or using tablet computers with the patient at the time of recruitment (or as soon thereafter as possible) and captures additional features not routinely recorded in the patient record including patient level of education, country of origin, smoking/alcohol history, social exclusion status (homelessness, injecting drug use, having been in prison), COVID-19 vaccination status, accommodation, employment status and travel history among other fields. Pre-morbid frailty status is assessed by the clinical research nurse using the clinical frailty score ^8^. Patient self-perceived social status is measured using the MacArthur Scale or ‘ladder score’ ^9, 10^.

Patient recovery is characterised using a case report form (Appendix C, Convalescent Case Report Form) completed at the time of clinical outpatient review, on paper or ideally directly on the clinical database. This includes back-to-work status, six-minute walk test results (conducted by our physiotherapy team) and Chalder fatigue scores. The six minute walk tests is an exercise **test** that measures distance walked over six minutes. It is a measure of functional status or fitness.

Clinical data is stored under a unique participant identifier on a bespoke STTAR Bioresource Clinical Data database on Dendrite, a secure electronic data capture (EDC) web platform for building and managing online databases and surveys. The Dendrite database operates on multiple-access levels and only the Principal Investigator (PI), Research Nurse and Clinical Research Fellow have complete access to the database and permission to modify. The STTAR Bioresource Clinical Data database is hosted by St James’s Hospital and security and access permissions are managed by the hospital’s information management system team. The database is protected behind host and institutional firewalls. The data controller for St. James’s Hospital patient data is St. James’s Hospital and for Tallaght University Hospital patient data held on the Dendrite database hosted by SJH, both institutions are data controllers. Inter institutional data sharing agreements between St. James’s Hospital, Tallaght University Hospital and Trinity College Dublin are in place.

Coded biological samples are processed and stored centrally at the STTAR Bioresource and archived by the biobank technician using industry standard Freezerworks software which only the biobank technician and lead study PI have access to. The registry database is shared with TCD IT service providers who manage their security and access permissions. This database is also periodically backed up on an external third-party server located in Dublin.

### Experimental data collection

TCD researchers who generate experimental data deriving from analysis of STTAR Bioresource samples or data, store this in dedicated TCD SharePoint folders, which is the preferred storage solution of the university. Each researcher has their own access-controlled folder. These data include spreadsheets (excel, CSV, .xlsx), Graphpad Prism files (.pzfx), flow cytometry files (.fcs), word documents, image files (.jpeg, .png, .ndpi), PowerPoint files (.pptx), transcriptomic data files (.cel). All coded experimental data pertaining to the STTAR Bioresource study is stored on TCD’s Microsoft OneDrive service. OneDrive is Microsoft's cloud-based file storage service, which allows syncing and sharing of files between computers and mobile devices and is the cloud computing service of choice of TCD. Data hosted on OneDrive is securely hosted by Microsoft in Europe in compliance with relevant legislation (see TCD Data Protection Procedural Guidelines).

## Ethical approval and safety measures

The STTAR Bioresource study has been granted ethical approval by the SJH/TUH Joint Research and Ethics Committee, (JREC 2020-05 List 19). This committee operates in compliance with and is constituted in accordance with the European Communities (Clinical Trials on Medicinal Products for Human Use) Regulations 2004 & ICH GCP guidelines. Health Research Consent Declaration Committee conditional approval was also obtained (HRCDC 20-012-AF1/COV). A data protection impact assessment and review was performed in each centre.

## Statistical analysis

Descriptive analysis of group characteristics includes number and proportion of cases for categorical variables, mean and standard deviation (SD) for normally distributed continuous variables, and median and inter-quartile range (IQR) for non-normally distributed continuous variables. When examining associations between COVID-19 disease severity and for example serum inflammatory markers, statistical methods are employed to account for confounding pre-existing diseases, e.g. multivariable logistic regression. Control groups of both vaccinated and unvaccinated age matched patients will be used to help validate results.

## Study governance ( Image 4)

**Image 4.**
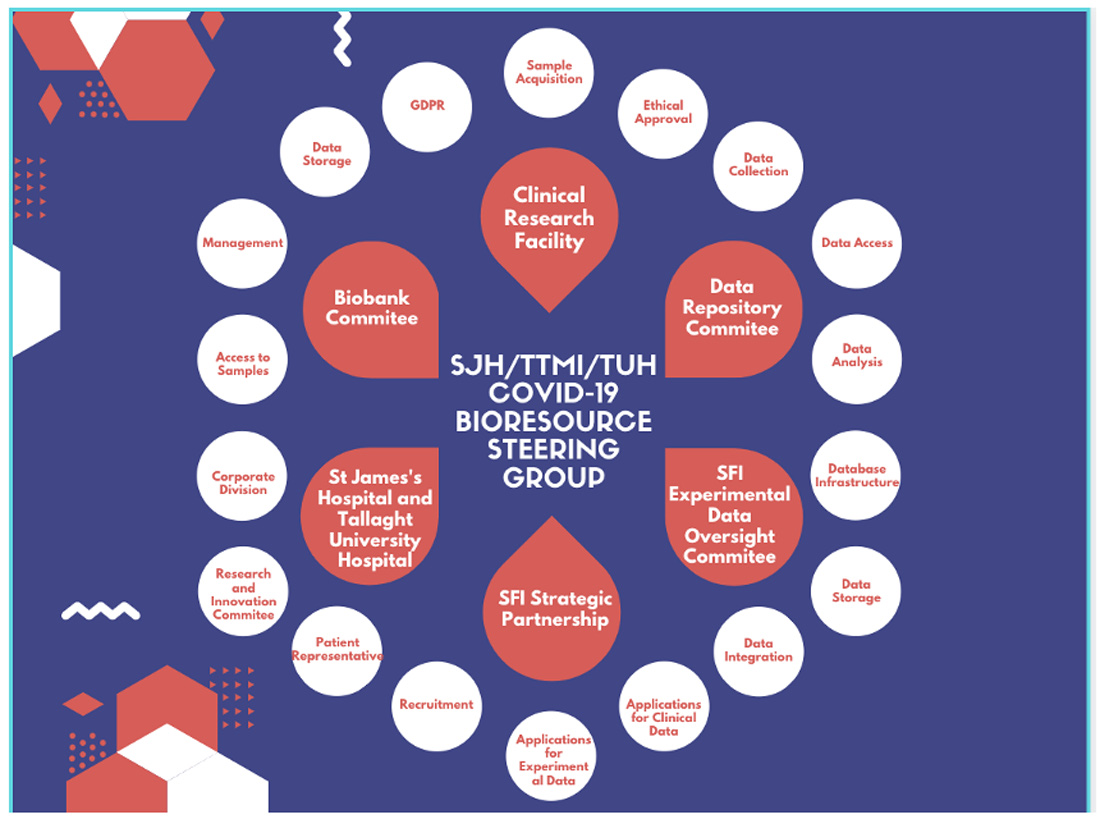
Clinical Governance. *Governance Structure. SFI = Science Foundation Ireland, SJH = St James’s Hospital, TUH = Tallaght University Hospital, TTMI = Trinity Translational Medicine Institute.*

## Patient and public involvement

Patient and public involvement (PPI) is an important and meaningful part of research ^11^. It has been argued that the profound and collective impact of the COVID-19 pandemic throughout society makes PPI more important than ever ^12, 13^. Accordingly, the authors have begun the process of recruiting patient representatives to the STTAR Bioresource committee. In addition, the STTAR Bioresource study will have a dedicated website to help disseminate its results to a wider audience.

## Study status

The STTAR Bioresource study continues to recruit new study participants from our two hospital sites as well as following up our existing participants. Studies looking at the relationship between socio-economic status and COVID-19 antibody levels post vaccination and another looking at the effects of COVID-19 infection on menstruation cycles of female participants are among the many projects underway at present. The steering committee of this study is continuing to welcome proposals and applications from researchers across our study sites.

## Dissemination

A fundamental mission of the STTAR Bioresource study is the timely and open-access dissemination of study results. This is achieved through publication of study results in open access journals and discussion at hospital grand rounds. Audits of data access are a fundamental part of this reporting procedure. All research outputs are reported in real time in open access formats and shared with the relevant stakeholders in line with the HRB joint statement on sharing research data and findings relevant to the novel coronavirus (COVID-19) outbreak. Detailed processes for data sharing, dissemination and exploitation are described in the data sharing agreement.

## Discussion

This research was prompted by a public health crisis that has impacted healthcare systems, economies, and societies globally. This study’s inclusive recruitment methods mean it involves patients from all socioeconomic backgrounds, all age groups, and all degrees of COVID-19 disease severity. The findings of the STTAR Bioresource study are relevant to policy and practice in the future care of patients with COVID-19 disease ^13– 15^.

The STTAR COVID Bioresource biobank was established despite limited financial and practical resources and has been, since its inception, reliant on the good will of St James’s (SJH) and Tallaght University Hospitals, Wellcome Trust Clinical Research Facility (SJH) and funding from Science Foundation Ireland as a Strategic Partnership Project (SFI-SPP).

Through large scale analysis of clinical, regional and genetic characteristics of COVID-19 patients, biobanks have helped identify, and so protect, at risk patient groups ^16, 17^. Twenty European Union states have come together to form the Biobanking and Biomolecular Resources Research Infrastructure – European Research Infrastructure Consortium (BBMRI-ERIC) ^18^. BBMRI-ERIC is a pan-European initiative providing one-stop access to the preserved biological samples of participating countries for research purposes. This pre-existing infrastructure allowed for the timely mobilisation of research communities across Europe in response to the COVID-19 pandemic ^19^. The lack of formal policy and procedures relating to biobanking in Ireland posed challenges to the establishment of a de novo COVID-19 biobank ^20^. There is currently no national biobank in Ireland and Ireland is not represented in BBMRI-ERIC. Calls for the establishment of a similar resource in Ireland conducive to high-impact research and data-sharing across research sites led to the announcement of funding of a National Irish COVID Biobank (NICB) by the HRB supported by Department of Health in July 2021 ^21^. The NICB will incorporate STTAR as well as other Irish COVID Biobanks.

This ambitious project will coordinate and harmonise data and sample collection across Irish sites and establish a unified governance framework in line with international biobanking standards. STTAR investigators have taken on leadership roles in this collaborative endeavour. As well as driving COVID-19 research at a national level, the complex process of setting up the NICB will inform non-cancer biobanking strategies in other disease areas into the future.

### Strengths of this study

Patients began being recruited to the study in April 2020, only short weeks after the first confirmed case of COVID-19 nationally. This early recruitment has allowed for an invaluable look at the first group of inpatients in Ireland with the disease. Over 1000 patients have been recruited to this study so far. This large study number increases the statistical value of any results. Detailed demographic data are being collected including patient occupation, number of people living in patients’ home, number of people sharing a bedroom with patient, home place (own accommodation, nursing home resident, homeless shelter resident) among other details. This allows us to analyse in detail a patient’s premorbid level of social deprivation, something not analysed in previous similar biobanks. The biobank participants are recruited from inpatient wards, intensive care units and outpatient staff groups. This allows for a wide range of disease severity (asymptomatic to critically unwell), disease acquisition (community, occupation and hospital-acquired) and age groups to be studied. Large numbers of healthcare workers are recruited to this study. This uniquely vulnerable group will be compared to the usual inpatient group. Looking more closely at infected health care workers may help inform future infection control procedures. Over 100 control patients have been recruited to the study. Vaccination status as well as usual case report form demographic details are collected. These healthy age matched control patients will allow comparison of results from studies conducted within STTAR Bioresource.

### Limitations of this study

Most patients who are enrolled in this study are hospital inpatients. Patients hospitalised with COVID-19 likely have severe disease requiring high level care. Recruiting most of our patients from the inpatient cohort means we may have a biased sample population and miss those with asymptomatic or mild disease. We follow our recruited patients in post COVID-19 outpatient clinics. Those who cancel their appointments due to resolution of symptoms are more difficult to capture in our study and so there may be a bias towards long COVID symptoms in those we see.

## Data availability

### Underlying data

No data are associated with this article.

### Extended data

Zenodo: Study Protocol for the St James's Hospital, Tallaght University Hospital, Trinity College Dublin Allied Researchers' (STTAR) Bioresource for COVID-19, https://doi.org/10.5281/zenodo.6278295.

This project contains the following extended data:

- Case Report Form, Informed Consent Form, Convalescent Report Form Online.docx

Data are available under the terms of the Creative Commons Attribution 4.0 International license (CC-BY 4.0).
