# Supplementary material for: Study protocol for the St James's Hospital, Tallaght University Hospital, Trinity College Dublin Allied Researchers' (STTAR) Bioresource for COVID-19
Source: HRB Open Res. 2022 Mar 16;5:20. [Version 1] doi: 10.12688/hrbopenres.13498.1 (PMC9111362; doi:10.12688/hrbopenres.13498.1)
Supplement: Peer review history

# Peer review: Reviewer response for version 1

**DOI:** 10.21956/hrbopenres.14722.r31787

**Published:** 2022-05-16

**Authors:** Mary Keogan

**Affiliations:** 1 Department of Clinical Immunology, Beaumont Hospital, Dublin, Ireland; 2 Royal College of Surgeons in Ireland, Dublin, Ireland

This paper describes the inception and rapid establishment of a collaborative biobanking resource, established within 2 months of the first confirmed COVID case worldwide. The collaboration involved 2 model 4, large teaching hospitals in Dublin, together with academic partners from Trinity College Dublin, and allied research institutes. This paper focuses on the steps undertaken to establish the biobank, and alludes to some of the research in progress which utilises this resource, but clearly, these individual studies will be disseminated in a specialty specific way.

 This study demonstrates the rapidity in which existing infrastructure can be mobilised to develop a high quality biobank, in response to a public health crisis, despite limited funding. The value of this biobank is greatly enhanced because of the early commencement of storage of material. The large range of disease severity, patient settings and detail of demographics and social factors additionally increase the value of this resource.

 This paper provides a systematic description of the processes involved in establishing a biobank, and will be of value to others considering the establishment of such a resource, regardless of disease area.

 There are ISO standards for Biobanking (ISO 20387:2018), and the need for compliance with such standards by funding and regulatory agencies is expected to increase over time. While the authors are to be congratulated for rapid mobilisation, and the effectiveness of the STTAR biobank to date, the relevance of this paper internationally would be enhanced by including a gap analysis of compliance with the relevant ISO standards.

Is the study design appropriate for the research question?

Yes

Is the rationale for, and objectives of, the study clearly described?

Yes

Are sufficient details of the methods provided to allow replication by others?

Yes

Are the datasets clearly presented in a useable and accessible format?

Not applicable

Reviewer Expertise:

Clinical Immunology; transplantation; Service delivery

I confirm that I have read this submission and believe that I have an appropriate level of expertise to confirm that it is of an acceptable scientific standard.

# Peer review: Reviewer response for version 1

**DOI:** 10.21956/hrbopenres.14722.r31688

**Published:** 2022-04-11

**Authors:** Luuk B. Hilbrands

**Affiliations:** 1 Department of Nephrology, Radboud University Medical Center (Radboudumc), Nijmegen, The Netherlands

This protocol describes a very well designed biobank and concurrent registry for COVID-19 patients, and healthy and disease specific controls.

 There is clear argumentation for the creation of the biobank and registry and the methods are described in sufficient detail.

 As recognized by the investigators, patients included in this project are mainly in-hospital patients and healthcare workers, which carries the risk of bias. If possible, it would be valuable to have access to national or regional registries of all COVID-19 patients to estimate the type and magnitude of selection bias. Similarly, the coupling of the study registry to a national registry of COVID-19 vaccination could provide more reliable data on the vaccination status of participants.

Is the study design appropriate for the research question?

Yes

Is the rationale for, and objectives of, the study clearly described?

Yes

Are sufficient details of the methods provided to allow replication by others?

Yes

Are the datasets clearly presented in a useable and accessible format?

Yes

Reviewer Expertise:

nephrology, immunology, kidney transplantation

I confirm that I have read this submission and believe that I have an appropriate level of expertise to confirm that it is of an acceptable scientific standard.
